# Selective activity of diselenides against *Aedes aegypti* (Diptera: Culicidae) larvae

**DOI:** 10.1590/0037-8682-0146-2020

**Published:** 2020-12-21

**Authors:** Sabrina Ketrin Targanski, Janaina Rosa Sousa, André Luiz Agnes Stein, Marcos Antônio Soares

**Affiliations:** 1 Universidade Federal de Mato Grosso, Departamento de Botânica e Ecologia, Cuiabá, MT, Brasil.; 2 Universidade Federal de Mato Grosso, Faculdade de Engenharia, Cuiabá, MT, Brasil.

**Keywords:** Selenium, 1,2-diphenyldiselenide, 1,2-bis(4-chlorophenyl) diselenide, Caenorhabditis elegans, Chlorella vulgaris, Galleria mellonela

## Abstract

**INTRODUCTION:**

*Aedes aegypti* (L.) is the major vector of arboviruses that causes serious public health concerns in tropical and subtropical countries.

**METHODS:**

We examined the larvicidal activity of 1,2-diphenyldiselenide [(PhSe)_2_] and 1,2-bis(4-chlorophenyl) diselenide [(p-ClPhSe)_2_] and determine its toxicity to different non-target organisms*.*

**RESULTS:**

(PhSe)_2_ and (p-ClPhSe)_2_ killed *Ae. aegypti* L3 larvae with LC_50/24h_ values of 65.63 µM (20.48 mg/L) and 355.19 µM (135.33 mg/L), respectively. (PhSe)_2_ was not toxic to the four model organisms.

**CONCLUSIONS:**

(PhSe)_2_ is a larvicidal compound with selective action against *Ae. aegypti* larvae. The mechanisms of action of (PhSe)_2_ under field conditions remain to be investigated.

*Aedes aegypti* (L.) is a mosquito widely distributed in tropical, subtropical, and temperate regions during warm seasons. The mosquito transmits human arboviruses, including dengue, chikungunya, and Zika virus^1^. It is estimated that the dengue virus infects over 390 million people in at least 100 countries on different continents and causes significant number of deaths and public health concerns. Zika virus infection has been associated with malformations in human fetal development^1^. The circulation of and co-infection by the dengue, chikungunya, and Zika viruses create a worrying scenario that demands effective measures to control the mosquito vector population^2^.

The *Ae. aegypti* populations can be controlled by different methods, such as the physical elimination of containers with larvae and pulp, chemical larvicides, and insecticides; the use of biological control agents such as *Bacillus thuringiensis* var. *israelensis* (Bti), *Wolbachia* bacteria, fish, and genetically modified male mosquitoes^2^. Larvicides are among the most important control agents because they eliminate immature forms and prevent the development of the adult form that transmits arboviruses. Bti is a larvicide of limited use because of its low persistence and the need for reapplication for effectiveness^3^. The chemical agent Pyriproxyfen acts more like a growth regulator than larvicide, and the agricultural pests *Bemisia tabaci* and *Musca domestica* (L.) are resistant to its action^4^. The development of new and efficient larvicides is important to broaden the arsenal of methods to control *Ae. aegypti* population. Larvicides must cause rapid larval death at low concentrations and no toxicity to non-target organisms. Sodium selenite suppresses the growth of *Culex quinquefasciatus* larvae and kills 50% of the larvae population at a concentration of 11 mg/L^5^. Selenium is a trace element essential for the antioxidant activity of selenoenzymes but can be toxic at high doses. Selenium compounds such as 1,2-diphenyldiselenide [(PhSe)_2_] exert various biological activities, including neuroprotective, anti-inflammatory, antiulcer, antidepressant, and anxiolytic activities. Its structural analog 1,2-bis(4-chlorophenyl) diselenide [(p-ClPhSe)_2_] prevents memory loss and treats metabolic disorders^6^. We hypothesize that low concentrations of selenium compounds (PhSe)_2_ and (p-ClPhSe)_2_ are toxic to *Ae. aegypti* larvae, but not other non-target organisms.

*Ae. aegypti* eggs were provided by Professor Margareth de Lara Capurro Guimarães, Ph.D., from the Institute of Biomedical Sciences of the University of São Paulo (USP), São Paulo, SP, Brazil. The eggs were hatched in glass jars containing autoclaved distilled water at 28 °C exposed to a 12 h photoperiod and fed with crushed fish feed (Tetramin^®^). Newly hatched larvae (L1) and third instar larvae (L3) were obtained approximately 12 h and 72 h after egg contact with water, respectively^7^.

The selenium compounds, (PhSe)_2_ and (p-ClPhSe)_2_, were synthesized according to a modified literature procedure^8^. The identity and purity of the compounds were confirmed by nuclear magnetic resonance (NMR) spectroscopy. ^1^H and ^13^C NMR spectra were recorded on a Bruker Ascend™ 500 MHz instrument with tetramethylsilane as an internal standard. Column chromatography was performed using Merck silica gel (230-400 mesh). Thin-layer chromatography was performed using Merck silica gel GF254 of 0.25 mm thickness, and the plates were revealed with either iodine vapor or acidic vanillin. (PhSe)_2_: yellow solid; yield: 74%. ^1^H NMR (CDCl_3_, 500 MHz) δ: 7.61 - 7.57 (m, 2H), 7.25 - 7.21 (m, 3H). ^13^C NMR (CDCl_3_, 125 MHz) δ: 127.2, 129.3, 131.1, 132.9. (p-ClPhSe)_2_: yellow solid; yield: 72%; ^1^H NMR (CDCl_3_, 500 MHz): δ = 7.53 (d, *J*= 8.6 Hz, 2H), 7.25 (d, *J*= 8.6 Hz, 2H); ^13^C NMR (CDCl_3_, 125 MHz) δ = 134.22, 133.18, 129.55, 129.29. Both compounds were dissolved in 10% dimethyl sulfoxide (DMSO) for biological assays.

The tests were performed in plastic cups (180 mL) containing 20 mL of tap water and 20 L3 of *Ae. Aegypti* ^7^, in the presence of different concentrations of (PhSe)_2_ and (p-ClPhSe)_2_ or 10% DMSO (control). The cups were incubated at 28°C with a 12 h photoperiod. The number of dead larvae were counted after 24 h and 48 h, and the larval mortality percentage was calculated. The (PhSe)_2_ and (p-ClPhSe)_2_ concentrations capable of killing 50% (LC_50_) and 90% (LC_90_) of the L3 larval population were estimated from the concentration-dependent mortality curves.

The eggs were kept in vials containing selenium compounds at a concentration corresponding to the LC_90/24h_ or 10% DMSO (control) to determine the death time of L1. The time from egg hatching to 100% L1 larvae death was recorded.

The toxicity of (PhSe)_2_ and (p-ClPhSe)_2_ to different model organisms was assessed at concentrations corresponding to the LC_90/24h_. The compounds’ environmental toxicity was estimated from the growth rate of the algae *Chlorella vulgaris* BR017 and the viability of the protozoan *Tetrahymena pyriformis*. The toxicity to non-target organisms was determined in *Caenorhabditis elegans* N2 and *Galleria mellonella* ^9^ *.*

Despite the availability of chemical control methods, the incidence of arboviruses transmitted by *Ae. aegypti*, such as dengue, chikungunya, and Zika, has increased annually, demanding the development of new larvicidal agents with low environmental toxicity and high selectivity and efficiency. Here, we report the selective larvicidal action of diphenyldiselenide (PhSe)_2_ against *Ae. aegypti* larvae and its low environmental impact.

The selenium compounds (PhSe)_2_ and (p-ClPhSe)_2_ killed *Ae. aegypti* L3 larvae and provided the LC_50/24h_ values of 65.63 µM (20.48 mg/L) and 355.19 µM (135.33 mg/L), respectively (Table 1). The World Health Organization proposes that promising larvicidal compounds are active at concentrations lower than 100 mg/L^7^. Compound (PhSe)_2_ fitted this criterion, as the concentrations required to kill 50% (LC_50/24h_), 90% (LC_90/24h_ = 108.14 µM or 33.74 mg/L), and 100% (160.25 µM or 50 mg/L) of L3 larvae within 24 h were lower than 100 mg/L (Table 1). Compounds (PhSe)_2_ and (p-ClPhSe)_2_ significantly differed (T-test, p <0.01) concerning the time required to kill *Ae. aegypti* L1 larvae: their LC_50/24h_ values were 32 ± 0 min and 380 ± 17.32 min, respectively.

**TABLE 1:** Concentration of selenium compounds capable of killing 50% (LC_50_) and 90% (LC_90_) of *Aedes aegypti* L3 larvae after 24 h and 48 h of treatment.

Parameter	(PhSe)_2_		(p-ClPhSe)_2_	
	µM	mg/L	µM	mg/L
LC_50/24h_	65.63	20.48	355.19	135.33
	(58.03-73.24)*	(18.10-22.85)	(313.99-396.4)	(119.63-151.03)
LC_90/24h_	108.14	33.74	602.28	229.47
	(96.96-129.26)	(30.25-40.33)	(540.71-712.24)	(206.01-271.36)
LC_50/48h_	52.44	16.36	191.72	73.04
	(46.9-57.98)	(14.63-18.09)	(164.52-218.92)	(62.68-83.41)
LC_90/48h_	84.54	26.38	393.42	14.89
	(76.65-98.46)	(23.91-30.72)	(345.76-474.6)	(131.735-180.82)

**(PhSe)**_2_ **:** 1,2-diphenyldiselenide; **(p-ClPhSe)**_2_ **:** 1,2-bis(4-chlorophenyl) diselenide. ^*^The values in parentheses represent the upper and lower limits at the 95% confidence interval.

N-substituted methyl maleamates, especially N-hexyl methyl maleamate at LC_50/24h_ = 0.36 mg/L (1.69 µM), probably affected *Ae. aegypti* larvae development through inhibition of sulfhydryl groups present in various key receptors essential for larval survival^10^. These compounds are efficient, but their toxicity has not been determined yet. Indole derivatives effectively control *Ae. aegypti* larvae population with low environmental toxicity, such as 6-bromo-2,3,4,9-tetrahydro-1H-carbazole with LC_50/24h_ = 1.5 mg/L (5.88 μM)^9^. Although the presence of chlorine or bromine groups in the aromatic ring usually improves the larvicidal activity of indole derivatives^9^, the addition of chlorine groups to (PhSe)_2_ to produce (p-ClPhSe)_2_ considerably reduced larvicidal activity (Table 1). The LC_50_ value of (PhSe)_2_ and its analogs in mice range from 278 to 381 mg/kg^11^. Compared with the control, treatment with (PhSe)_2_ at LC_90/24h_ (108.14 µM or 33.74 mg/L) did not alter *C. elegans* and *G. mellonella* survival rate. At the same concentration, (PhSe)_2_ did not affect: (i) *Chlorella vulgaris* BR017 cell growth rate (1.15×10^6^ ± 4.02×10^5^ cells/mL) when compared with the control (7.83×10^5^ ± 1.54×10^5^ cells/mL) on the seventh analysis (T-test, p <0.01); and (ii) *Tetrahymena* sp. viability (1.53×10^6^ ± 5.05×10^4^ cells/mL) when compared with the control (1.0×10^6^ ± 2.0×10^4^ cells/mL) (T-test, p <0.01). Therefore, (PhSe)_2_ was not toxic to the four organisms studied, at least under the conditions assessed. Diorganoyldiselenides - (PhSe)_2_ and (p-ClPhSe)_2_ - reduce food intake in animals, resulting in a dose-dependent decrease in body weight^12^. The larvicidal activity of (PhSe)_2_ in *Ae. aegypti* larvae may be associated with the free radical generation and oxidation of thiol groups that usually mediate these compounds’ toxicity to mammals^13^ ^,^ ^14^ and *Drosophila melanogaster* ^15^. Our findings help to develop larvicidal products from (PhSe)_2_ with selective action against *Ae. aegypti* larvae and low environmental toxicity, but their mechanisms of action remain to be determined.
